# Mortality trends in people with disabilities before and during the COVID-19 pandemic in South Korea, 2017–2022

**DOI:** 10.3389/fpubh.2024.1414515

**Published:** 2024-07-25

**Authors:** Ye-Soon Kim, Ju-Hee Kim, Sooyoung Kwon, Joo-Hee Kim, Hyun-Ji Kim, Seung Hee Ho

**Affiliations:** Department of Healthcare and Public Health Research, Korea National Rehabilitation Center, Rehabilitation Research Institute, Seoul, Republic of Korea

**Keywords:** COVID-19, mortality, people with disability, South Korea, infection

## Abstract

**Objective:**

To investigate temporal trends in mortality rates and underlying causes of death in persons with disabilities before and during the coronavirus disease 2019 (COVID-19) pandemic.

**Methods:**

Annual mortality rates and causes of death were analyzed using data covering the 2017–2022 period.

**Results:**

The mortality rate among people with disabilities increased from 2017 to 2022; the rate was five times higher during COVID-19 in this population than in the general population. When analyzing the cause of death, the incidence of infectious diseases and tuberculosis decreased after COVID-19. In contrast, the incidence of other bacillary disorders (A30–A49) increased. The incidence of respiratory system diseases (J00–J99), influenza and pneumonia (J09–J18), and other acute lower respiratory infections (J20–J22) decreased before COVID-19, while the incidence of lung diseases due to external agents (J60–J70), other respiratory diseases principally affecting the interstitium (J80–J84), and other diseases of the pleura (J90–J94) increased during the pandemic. The risk of COVID-19 death among people with disabilities was 1.1-fold higher for female patients (95% CI = 1.06–1.142), 1.41-fold for patients aged 70 years and older (95% CI = 1.09–1.82), and 1.24-fold higher for people with severe disabilities (95% CI = 1.19–1.28).

**Conclusions:**

The mortality rate in people with disabilities significantly increased during COVID-19, compared with that before the pandemic. People with disabilities had a higher mortality rate during COVID-19 compared with the general population. Risk factors must be reduced to prevent high mortality rates in this population.

## 1 Introduction

On January 30, 2020, the World Health Organization (WHO) declared a public health emergency due to international concern over the outbreak of coronavirus disease 2019 (COVID-19) (1). South Korea began social distancing on February 29, 2020, and on March 22, 2020, it began to impose more substantial social distancing measures (2). Restrictions on the use of religious facilities and the operation of commercial industries were affected. Over time, social distancing was initiated and suspended several times (3). On May 5, 2023, the WHO declared that COVID-19 was no longer an international public health emergency. Consequently, on May 11, 2023, Korean health authorities lifted most of the COVID-19 quarantine measures, declaring it an infectious disease that has become endemic (4).

The mortality rate related to the COVID-19 pandemic was not constant across population subsets. Older people (5–7), women (8, 9), ethnic minorities, and particular population groups (10, 11) were more likely to die from COVID-19. This information helped in the COVID-19 response and may help in responding to future infectious diseases. Moreover, this information was applied in policies involving social distancing (12, 13), allotting vaccination priority (14, 15), and quarantine and public relations (16–18).

In South Korea, men had a higher COVID-19-related mortality rate than women (19). Among patients with COVID-19, older individuals had higher case fatality and symptomatic infection rates, leading to a high mortality rate. In Korea, approximately 80% of COVID-19-related deaths occurred in people aged over 70 years (20). However, epidemiological indicators such as infection rate, hospitalization, mortality rate, and cause of death have not been separately evaluated for people with disabilities, who are expectedly vulnerable.

As such, there is insufficient information on the current status and risk of COVID-19 mortality in people with disabilities. Reports of mortality exist, although these are limited to some types of disabilities such as physical disabilities and developmental and mental disorders. However, there is an apparent lack of data on COVID-19-related mortality rate and mortality risk encompassing all people with disabilities. Therefore, we aimed to answer the following research questions: (1) Has the pandemic brought about a change in the mortality rate and cause of death in people with disabilities? (2) Is the pandemic-related mortality rate in people with disabilities similar to that in the general population?

## 2 Materials and methods

### 2.1 Data sources and study population

A death cause database was created by linking Statistics Korea data on the cause of death with the data of registered people with disabilities from the Ministry of Health and Welfare in South Korea. Registered people with disabilities are subject to the Korean Emotional Welfare Act (Act on Welfare of Persons with Disabilities) of the, Ministry of Government Legislation (21), which is legally registered with the Korean government (approximately 265 million registered people). There are 15 types of disabilities, each divided into severe and mild (22). The database records the number of registered people with disabilities nationwide. The data obtained for this study included sex, age, main disability type, and degree of disability. Any missing item resulted in exclusion from the analysis. Cause of death was classified using the International Classification of Diseases, Tenth Revision (ICD-10) codes and recorded on the patients' death certificates. This falls within the scope of the World Health Organization's 103 items. This study was approved by the Institutional Review Board of the National Rehabilitation Institute (approval number: NRC-2023-01-008).

### 2.2 Statistical analysis

Crude mortality and age-standardized mortality rates were calculated using death data for persons with disabilities in South Korea. The crude death rate for people with disabilities is an indicator calculated by dividing the total number of deaths of registered people with disabilities that occurred in a year by the estimated population of the country for that year, expressed per 100,000 people. The number of deaths of registered people with disabilities is collected at the end of the relevant year. The population of registered people with disabilities was operationally defined per the following formula:


$$\begin{array}{l} \frac{Number\;of\;registered\;people\;with\;disabilities\;in\;previous\;year+Number\;of\;registered\;people\;with\;disabilities\;in\;current\;year}{2} \end{array}$$

The overall population crude death rate was set to the same standard as the crude death rate for people with disabilities. However, this figure includes people with and without disabilities. Logistic regression analysis was used to analyze factors affecting COVID-19 deaths in people with disabilities. The significance level was set at p < 0.05. The data were analyzed with SAS 9.4 statistical software (SAS Institute Inc, Cary, NC).

## 3 Results

### 3.1 Changes in mortality rates among people with disabilities before and after the COVID-19 pandemic

**Figure 1:**
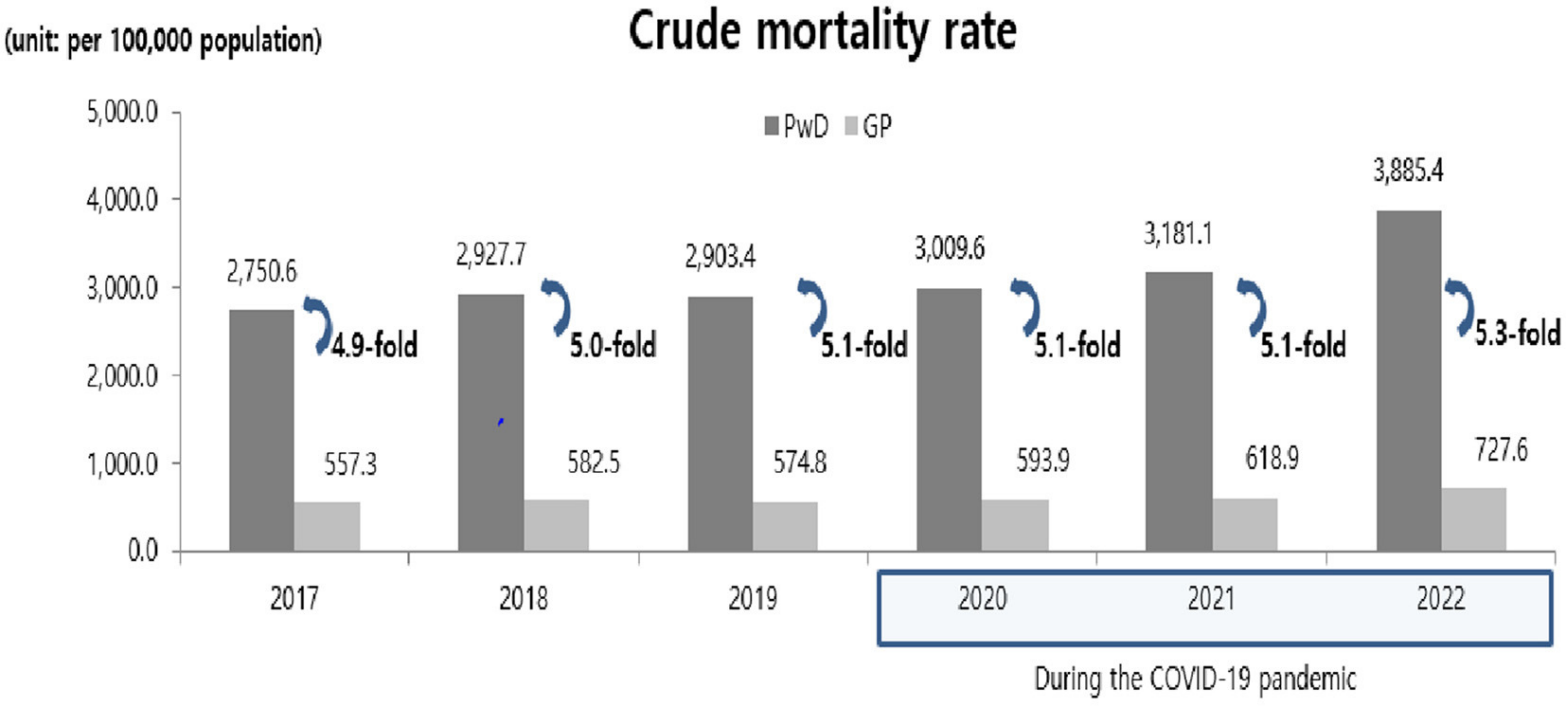
Mortality trends among people with and without disabilities before and during the COVID-19 pandemic. PwD, People with disabilities; GP, General population; COVID-19, coronavirus disease 2019.

**Figure 2:**
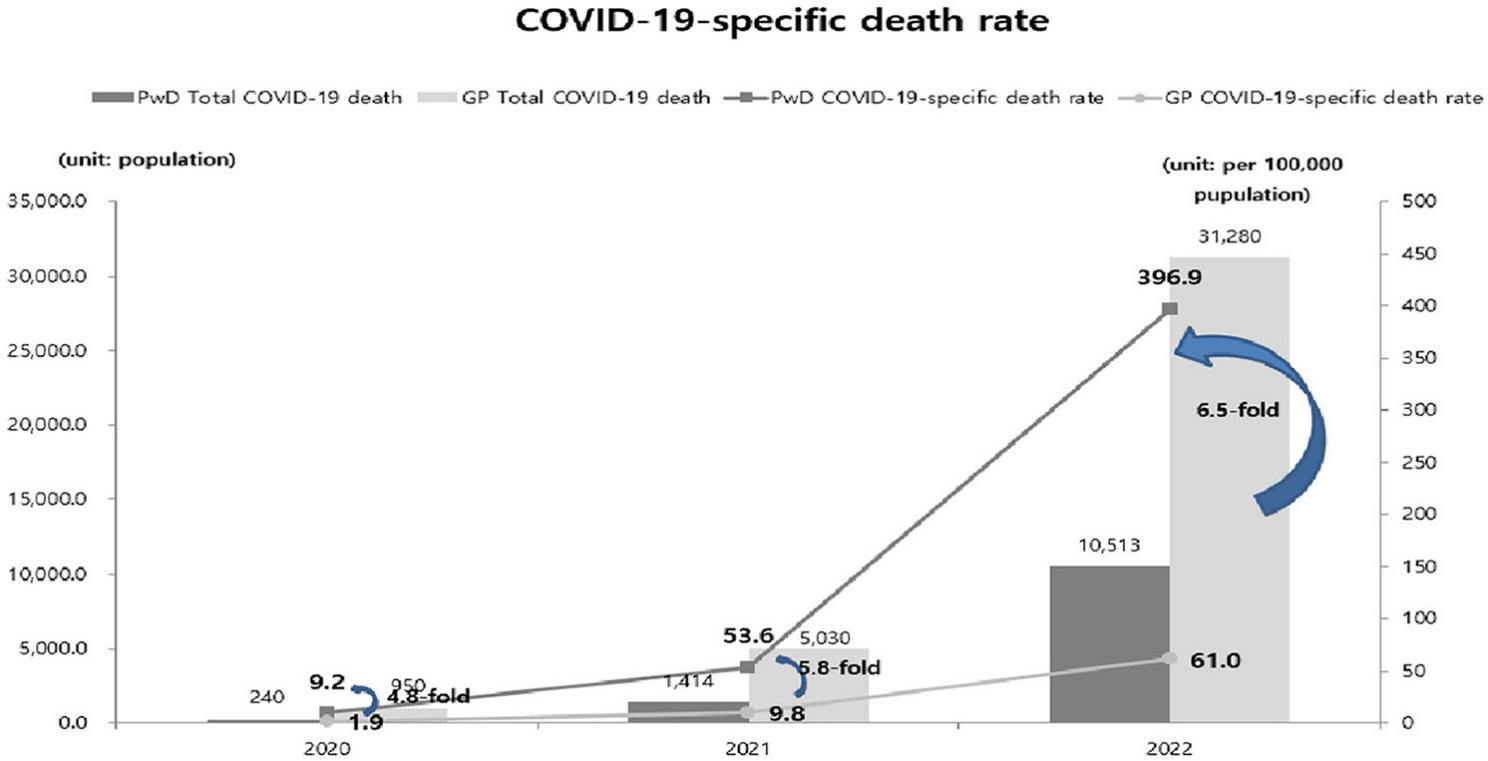
COVID-19 specific death rate trends among people with and without disabilities during the COVID-19 pandemic. COVID-19, coronavirus disease 2019; PwD, People with disabilities; GP, General population.

Figure 1 shows the changes in the mortality rates of people with disabilities before and after the COVID-19 pandemic. From 2017 to 2022, the number of deaths among people with disabilities increased. Concurrently, the crude mortality rate showed an overall increase. The general population showed the same pattern as that of people with disabilities. Over the past 5 years, mortality rates have increased. In 2020, there were 240 deaths among people with disabilities due to COVID-19, increasing to 1,414 in 2021 and 10,513 in 2022. Additionally, COVID-19-related mortality increased from 9.2 to 396.9. The mortality rate gap between people with disabilities and the general population increased 4.8-fold in 2020, 5.5-fold in 2021, and 6.5-fold in 2022 (Figure 2).

### 3.2 Changes in cause of death among people with disabilities before and after the COVID-19 pandemic

Table 1 shows changes in ICD-10 codes for cause of death among people with disabilities during the pre- and post-COVID-19 period. The number of deaths among people with disabilities increased over the 5-years study period. Between 2020 and 2022, significant increases were observed in symptoms, signs, and abnormal clinical and laboratory findings not elsewhere classified (NEC, R00–R99), and in the provisional assignment of new diseases of uncertain etiology or emergency use (U00–U18).

**Table 1:** Causes of death among people with disabilities based on ICD-10 codes from 2017 to 2022.

**ICD-10 categories**	**2017 (*****N*** = **69,478)**			**2018 (*****N*** = **74,957)**			**2019 (*****N*** = **75,402)**			**2020 (*****N*** = **78,879)**			**2021 (*****N*** = **83,858)**			**2022 (*****N*** = **102,916)**		
	***n* (%)**	**CSMR**	**SMR**	***n* (%)**	**CSMR**	**SMR**	***n* (%)**	**CSMR**	**SMR**	***n* (%)**	**CSMR**	**SMR**	***n* (%)**	**CSMR**	**SMR**	***n* (%)**	**CSMR**	**SMR**
A00–B99	2,178 (3.13)	86.2	24.7	2,565 (3.42)	100.2	27.7	2,562 (3.40)	98.7	25.6	2,953 (3.74)	112.7	31.4	3,154 (3.76)	119.6	27.2	3,254 (3.16)	122.8	28.3
C00–D48	14,436 (20.78)	571.5	167.7	14,886 (19.86)	581.4	162.5	15,715 (20.84)	605.1	165.7	15,981 (20.26)	609.7	161.8	16,353 (19.50)	620.3	155.0	16,616 (16.15)	627.3	162.4
D50–D89	179 (0.26)	7.1	2.2	214 (0.29)	8.3	3.4	206 (0.27)	7.9	3.3	224 (0.28)	8.5	3.8	223 (0.27)	8.5	2.5	218 (0.21)	8.2	4.2
E00–E90	3,824 (5.50)	151.4	66.0	3,972 (5.30)	155.1	43.2	3,589 (4.76)	138.2	39.5	3,766 (4.77)	143.7	39.7	3,869 (4.61)	146.8	42.2	4,953 (4.81)	187.0	52.1
F00–F99	1,155 (1.66)	45.7	11.3	1,089 (1.45)	42.5	9.8	1,077 (1.43)	41.5	8.9	1,011 (1.28)	38.6	9.8	886 (1.06)	33.6	8.5	927 (0.90)	35.0	9.6
G00–G99	3,854 (5.55)	152.6	89.3	4,366 (5.82)	170.5	96.0	4,290 (5.69)	165.2	84.9	4,643 (5.89)	177.2	73.4	5,085 (6.06)	192.9	84.2	6,520 (6.34)	246.1	101.0
H00–H59	0 (0.00)	NA	NA	4 (0.01)	0.2	0.1	1 (0.00)	0.04	0.005	0 (0.00)	NA	NA	0 (0.00)	NA	NA	0 (0.00)	NA	NA
H60–H95	3 (0.00)	0.1	0.01	6 (0.01)	0.2	0.04	5 (0.01)	0.2	0.02	2 (0.00)	0.08	0.007	6 (0.01)	0.2	0.2	5 (0.00)	0.2	0.017
I00–I99	17,592 (25.32)	696.5	162.8	18,396 (24.54)	718.5	163.3	17,795 (23.60)	685.2	154.6	18,613 (23.60)	710.2	150.9	18,920 (22.56)	717.7	143.9	21,550 (20.94)	813.6	157.5
J00–J99	9,441 (13.59)	373.8	82.3	11,181 (14.92)	436.7	91.5	11,364 (15.07)	437.6	87.4	11,315 (14.34)	431.7	82.3	11,648 (13.89)	441.9	77.3	13,368 (12.99)	504.7	92.8
K00–K93	2,760 (3.97)	109.3	38.7	2,888	112.8	42.2	2,887 (3.83)	111.2	39.4	3,083 (3.91)	117.6	41.1	3,200 (3.82)	121.4	42.3	3,535 (3.43)	133.5	47.2
L00–L99	191 (0.27)	7.6	1.7	207 (0.28)	8.1	1.7	188 (0.25)	7.2	1.6	224 (0.28)	8.5	2.2	191 (0.23)	7.2	1.7	186 (0.18)	7.0	1.5
M00–M99	441 (0.63)	17.5	5.7	429 (0.57)	16.8	4.8	447 (0.59)	17.2	5.1	458 (0.58)	17.5	4.8	463 (0.55)	17.6	5.0	547 (0.53)	20.7	5.8
N00–N99	3,376 (4.86)	133.7	34.6	3,644 (4.86)	142.3	36.1	3,989 (5.29)	153.6	34.7	4,223 (5.35)	161.1	37.1	4,411 (5.26)	167.3	35.7	4,754 (4.62)	179.5	41.3
O00–O99	2 (0.00)	0.1	0.2	0 (0.00)	NA	NA	1 (0.00)	0.04	0.2	1 (0.00)	0.04	0.1	0 (0.00)	NA	NA	2 (0.00)	0.1	0.3
P00–P96	2 (0.00)	0.1	1.6	1 (0.00)	0.04	0.8	2 (0.00)	0.08	1.4	1 (0.00)	0.04	0.2	3 (0.00)	0.1	1.8	7 (0.01)	0.3	3.9
Q00–Q99	112 (0.16)	4.4	18.3	106 (0.14)	4.1	14.8	110 (0.15)	4.2	14.6	112 (0.14)	4.3	17.9	116 (0.14)	4.4	14.3	129 (0.13)	4.9	21.1
R00–R99	5,424 (7.81)	214.7	53.7	6,312 (8.42)	246.5	59.3	6,583 (8.73)	253.5	59.8	7,448 (9.44)	284.2	60.4	9,449 (11.27)	358.4	71.6	11,077 (10.76)	418.2	77.1
U00–U18	0 (0.00)	NA	NA	0 (0.00)	NA	NA	0 (0.00)	NA	NA	240 (0.30)	9.2	1.7	1,414 (1.69)	53.6	12.3	10,513 (10.22)	396.9	85.3
V01–Y98	4,507 (6.49)	178.4	93.0	4,691 (6.26)	183.2	90.8	4,591 (6.09)	176.8	89.7	4,581 (5.81)	174.8	91.9	4,467 (5.33)	169.5	77.4	4,755 (4.62)	179.5	81.6

A00–B99 (certain infectious and parasitic diseases); C00–D48 (neoplasms); D50–D89 (diseases of the blood and blood-forming organs and certain disorders involving the immune mechanism); E00–E90 (endocrine, nutritional, and metabolic diseases); F00–F99 (mental and behavioral disorders); G00–G99 (diseases of the nervous system); H00–H59 (diseases of the eye and adnexa); H60–H95 (diseases of the ear and mastoid process); I00–I99 (diseases of the circulatory system); J00–J99 (diseases of the respiratory system) K00–K93 (diseases of the digestive system); L00–L99 (diseases of the skin and subcutaneous tissue); M00–M99 (diseases of the musculoskeletal system and connective tissue); N00–N99 (diseases of the genitourinary system); O00–O99 (pregnancy, childbirth, and puerperium); P00–P96 (certain conditions originating in the perinatal period); Q00–Q99 (congenital malformations, deformations, and chromosomal abnormalities); R00–R99 (symptoms, signs, and abnormal clinical and laboratory findings, and NEC); U00–U18 (provisional assignment of new diseases of uncertain etiology or emergency use); V01–Y98 (external causes of morbidity and mortality).

n, number of deaths; ICD-10, International Classification of Diseases, Tenth Revision; CSMR, cause-specific mortality rate (per 100,000 people); SMR; age-adjusted standardized mortality rate (per 100,000 people); NA, Not Available; NEC, not elsewhere classified.

### 3.3 Changes in cause of death related to COVID-19 in people with disabilities after the COVID-19 pandemic

Three disease types with significant changes between 2019 and 2020 before and after COVID-19 were observed in people with disabilities: Diseases of the nervous system (G00–G99), certain infectious and parasitic diseases (A00–B99), and diseases of the respiratory system (J00–J99) on which an intensive study was conducted. These results are presented in Tables 2–**4**.

**Table 2:** Causes of death among people with disabilities by ICD-10-based diseases of the nervous system (G00–G99) from 2017 to 2022.

**Diseases of the nervous system (G00–G99)**	**2017 (*N* = 3,854)**	**2018 (*N* = 4,366)**	**2019 (*N* = 4,290)**	**2020 (*N* = 4,643)**	**2021 (*N* = 5,085)**	**2022 (*N* = 6,520)**
	***n* (%)**	***n* (%)**	***n* (%)**	***n* (%)**	***n* (%)**	***n* (%)**
Inflammatory diseases of the central nervous system (G00–G09)	55 (1.43)	69 (1.58)	72 (1.68)	63 (1.36)	71 (1.40)	90 (1.38)
Systemic atrophies primarily affecting the central nervous system (G10–G14)	408 (10.59)	432 (9.89)	409 (9.53)	370 (7.97)	409 (8.04)	496 (7.61)
Extrapyramidal and movement disorders (G20–G26)	1,564 (40.58)	1,749 (40.06)	1,490 (34.73)	1,589 (34.22)	1,769 (34.79)	2,070 (31.75)
Other degenerative diseases of the nervous system (G30–G32)	1,043 (27.06)	1,318 (30.19)	1,501 (34.99)	1,811 (39.00)	1,935 (38.05)	2,789 (42.78)
Demyelinating diseases of the central nervous system (G35–G37)	16 (0.42)	22 (0.50)	14 (0.33)	21 (0.45)	13 (0.26)	24 (0.37)
Episodic and paroxysmal disorders (G40–G47)	224 (5.81)	238 (5.45)	231 (5.38)	220 (4.74)	251 (4.94)	312 (4.79)
Nerve, nerve root, and plexus disorders (G50–G59)	1 (0.03)	1 (0.02)	0 (0.00)	2 (0.04)	5 (0.10)	3 (0.05)
Polyneuropathies and other disorders of the peripheral nervous system (G60–G64)	14 (0.36)	18 (0.41)	11 (0.26)	13 (0.28)	25 (0.49)	22 (0.34)
Diseases of myoneural junction and muscle (G70–G73)	132 (3.43)	133 (3.05)	117 (2.73)	114 (2.46)	119 (2.34)	119 (1.83)
Cerebral palsy and other paralytic syndromes (G80–G83)	149 (3.87)	144 (3.30)	156 (3.64)	126 (2.71)	114 (2.24)	190 (2.91)
Other disorders of the nervous system (G90–G99)	248 (6.43)	242 (5.54)	289 (6.74)	314 (6.76)	374 (7.35)	405 (6.21)

n, number of deaths; ICD-10, International Classification of Diseases, Tenth Revision.

#### 3.3.1 Diseases of the nervous system (G00–G99)

From 2017 to 2020, the number of deaths among people with disabilities due to diseases of the nervous system (G00–G99) increased. This number further increased during COVID-19, from 2020 to 2022. It is difficult to clearly observe the change in mortality rates among people with disabilities due to diseases of the nervous system (G00–G99) after COVID-19. However, the diseases that caused an increase in the number of deaths since 2020 compared with 2019 included extrapyramidal and movement disorder (G20–G26) and other generic disorders of the nervous system (G30–G32) (Table 2).

#### 3.3.2 Certain infectious and parasitic diseases (A00–B99)

The number of individuals with certain infectious and parasitic diseases (A00-B99) among people with disabilities was 2,953 in 2020, 3,151 in 2021, and 3,254 in 2022, showing an increasing trend since COVID-19. However, the incidence of infectious intestinal diseases and tuberculosis decreased after COVID-19. In contrast, the incidence of other bacillary disorders (A30–A49) increased (Table 3).

**Table 3:** Causes of death among people with disabilities by ICD-10-based certain infectious and parasitic diseases (A00–B99) from 2017 to 2022.

**Certain infectious and parasitic diseases (A00–B99)**	**2017 (*N* = 2,178)**	**2018 (N =,2,565)**	**2019 (*N* = 2,562)**	**2020 (*N* = 2,953)**	**2021 (*N* = 3,154)**	**2022 (*N* = 3,254)**
	***n* (%)**	***n* (%)**	***n* (%)**	***n* (%)**	***n* (%)**	***n* (%)**
Intestinal infectious diseases (A00–A09)	265 (12.17)	301 (11.73)	282 (11.01)	301 (10.19)	297 (9.42)	310 (9.53)
Tuberculosis (A15–A19)	393 (18.04)	412 (16.06)	359 (14.01)	351 (11.89)	368 (11.67)	336 (10.33)
Certain zoonotic bacterial diseases (A20–A28)	0 (0.00)	0 (0.00)	0 (0.00)	0 (0.00)	0 (0.00)	1 (0.03)
Other bacterial diseases (A30–A49)	1,262 (57.94)	1,558 (60.74)	1,625 (63.43)	2,036 (68.95)	2,251 (71.37)	2,318 (71.24)
Infections with a predominantly sexual mode of transmission (A50–A64)	0 (0.00)	1 (0.04)	1 (0.04)	2 (0.07)	3 (0.10)	2 (0.06)
Other spirochetal diseases (A65–A69)	0 (0.00)	0 (0.00)	0 (0.00)	1 (0.03)	0 (0.00)	0 (0.00)
Other diseases caused by chlamydia (A70–A74)	0 (0.00)	2 (0.08)	1 (0.04)	3 (0.10)	5 (0.16)	0 (0.00)
Rickettsioses (A75–A79)	9 (0.41)	0 (0.00)	0 (0.00)	0 (0.00)	0 (0.00)	13 (0.04)
Viral infections of the central nervous system (A80–A89)	19 (0.87)	16 (0.62)	13 (0.51)	9 (0.30)	21 (0.67)	20 (0.61)
Arthropod-borne viral fevers and viral hemorrhagic fevers (A92–A99)	6 (0.28)	4 (0.16)	10 (0.39)	8 (0.27)	4 (0.13)	7 (0.22)
Viral infections characterized by skin and mucous membrane lesions (B00–B09)	4 (0.18)	7 (0.27)	8 (0.31)	10 (0.34)	5 (0.16)	10 (0.31)
Viral hepatitis (B15–B19)	114 (5.23)	138 (5.38)	159 (6.21)	125 (4.23)	101 (3.20)	108 (3.32)
Human immunodeficiency virus [HIV] disease (B20–B24)	17 (0.78)	16 (0.62)	9 (0.35)	6 (0.20)	6 (0.19)	7 (0.22)
Other viral diseases (B25–B34)	2 (0.09)	7 (0.27)	4 (0.16)	7 (0.24)	5 (0.16)	7 (0.22)
Mycoses (B35–B49)	21 (0.96)	38 (1.48)	28 (1.09)	37 (1.25)	55 (1.74)	72 (2.21)
Protozoal diseases (B50–B64)	6 (0.28)	12 (0.47)	8 (0.31)	15 (0.51)	0 (0.00)	0 (0.00)
Helminthiases (B65–B83)	2 (0.09)	0 (0.00)	1 (0.04)	2 (0.07)	2 (0.06)	1 (0.03)
Pediculosis, acariasis, and other infestations (B85–B89)	0 (0.00)	0 (0.00)	2 (0.08)	2 (0.07)	0 (0.00)	0 (0.00)
Sequelae of infectious and parasitic diseases (B90–B94)	54 (2.48)	46 (1.79)	50 (1.95)	29 (0.98)	21 (0.67)	37 (1.14)
Bacterial, viral, and other infectious agents (B95–B98)	0 (0.00)	0 (0.00)	0 (0.00)	0 (0.00)	0 (0.00)	0 (0.00)
Other infectious diseases (B99)	4 (0.18)	7 (0.27)	2 (0.08)	9 (0.30)	10 (0.32)	5 (0.15)

n, number of deaths; ICD-10, International Classification of Diseases, Tenth Revision.

#### 3.3.3 Diseases of the respiratory system (J00–J99)

In the 5-year study period, the incidence of diseases of the respiratory system (J00-J99) among people with disabilities increased. Particularly, in 2021, 11,648 people with disabilities were newly diagnosed with diseases of the respiratory system; more than those diagnosed in the 2017–2020 period. The incidence of influenza and pneumonia (J09–J18), other acute lower respiratory infections (J20–J22), and diseases of the respiratory system decreased after COVID-19. Contrarily, the incidence of lung diseases due to external agents (J60–J70), other respiratory diseases principally affecting the interstitium (J80–J84), other diseases of the pleura (J90–J94), and other diseases of the respiratory system (J95–J99) increased after COVID-19 (Table 4).

**Table 4:** Causes of death among people with disabilities by ICD-10-based diseases of the respiratory system (J00-J99) from 2017 to 2022.

**Diseases of the respiratory system (J00–J99)**	**2017 (*N* = 9,441)**	**2018 (*N* = 11,181)**	**2019 (*N* = 11,364)**	**2020 (*N* = 11,315)**	**2021 (*N* = 11,648)**	**2022 (*N* = 13,368)**
	***n* (%)**	***n* (%)**	***n* (%)**	***n* (%)**	***n* (%)**	***n* (%)**
Acute upper respiratory infections (J00–J06)	9 (0.10)	9 (0.08)	5 (0.04)	6 (0.05)	2 (0.02)	8 (0.06)
Influenza and pneumonia (J09–J18)	5,681 (60.17)	7,075 (63.28)	7,253 (63.82)	7,126 (62.98)	7,247 (62.22)	8,626 (64.53)
Other acute lower respiratory infections (J20–J22)	18 (0.19)	13 (0.12)	12 (0.11)	10 (0.09)	10 (0.09)	12 (0.09)
Other diseases of the upper respiratory tract (J30–J39)	10 (0.11)	16 (0.14)	9 (0.08)	10 (0.09)	16 (0.14)	12 (0.09)
Chronic lower respiratory diseases (J40–J47)	1,947 (20.62)	1,949 (17.43)	1,953 (17.19)	1,726 (15.25)	1,707 (14.65)	1,950 (14.59)
Lung diseases due to external agents (J60–J70)	916 (9.70)	1,080 (9.66)	1,079 (9.49)	1,300 (11.49)	1,350 (11.59)	1,315 (9.84)
Other respiratory diseases principally affecting the interstitium (J80–J84)	477 (5.05)	534 (4.78)	540 (4.75)	542 (4.79)	637 (5.47)	732 (5.48)
Suppurative and necrotic conditions of the lower respiratory tract (J85–J86)	70 (0.74)	88 (0.79)	90 (0.79)	81 (0.72)	96 (0.82)	86 (0.64)
Other diseases of the pleura (J90–J94)	54 (0.57)	77 (0.69)	72 (0.63)	75 (0.66)	107 (0.92)	94 (0.70)
Other diseases of the respiratory system (J95–J99)	259 (2.74)	340 (3.04)	351 (3.09)	439 (3.88)	476 (4.09)	533 (3.99)

n, number of deaths; ICD-10, International Classification of Diseases, Tenth Revision.

### 3.4 Analysis of COVID-19-related deaths among people with disabilities from 2020 to 2022

As a multivariate analysis of COVID-19 deaths in people with disabilities, binomial logistic regression analysis was performed, and the odds ratio was calculated. Compared with males, females were significantly more likely to die from COVID-19 (*p* < 0.05). Compared with individuals under 30 years of age, the probability of death was 0.894-fold lower for those between 30 and 49 years of age, which was statistically significant (*p* = 0.0015). Compared with patients under the age of 30 years, those aged ≥ 70 years had a 1.41-fold higher probability of death, showing statistical significance. The risk of COVID-19 death in people with severe disabilities was 1.24-fold higher than that in those with mild disabilities, which was statistically significant (Table 5).

**Table 5:** Binomial logistic regression analysis of COVID-19 deaths among people with disabilities from 2020 to 2022.

**Variable**	**Category**	***p***	**OR**	**95% CI**	
Sex	Male		1.000		
	Female	0.0000	1.101	1.061	1.142
Age group (years)	< 30		1		
	30–49	0.0015	0.894	0.670	1.193
	50–69	0.6327	1.110	0.858	1.437
	≥70	0.0000	1.417	1.098	1.829
Degree of disability	Mild		1		
	Severe	0.0000	1.240	1.195	1.287

COVID-19, coronavirus disease 2019; OR, Odds ratio; CI, Confidence interval.

p-value was calculated by binomial logistic regression.

## 4 Discussion

A database was established by linking data for registered people with disabilities in Korea with data from the Korea National Statistical Office (2017–2022), and mortality rate and cause of death were analyzed using the death database. The analysis of our findings in the context of previous studies is discussed below.

First, the number of deaths among people with disabilities due to COVID-19 increased in 2022 compared with 2020. The mortality rate due to COVID-19 increased from 9.2 people in 2020 to 396.9 people in 2022. The related gap between people with disabilities and the general population was 4.8-fold higher in 2020 and 6.5-fold higher in 2022 (23). According to a study by Kuper et al., the combined adjusted effect estimate for COVID-19-related mortality in people with disabilities compared with those without disabilities was 2.7-fold (95% confidence interval, 2.4–3.2) (24). The study highlighted that people with disabilities had a higher COVID-19-related mortality than the general population (25), despite the differences in degree of disability and country.

Second, there was a change in the causes of death during COVID-19 among people with disabilities, compared with that before COVID-19. During the COVID-19 pandemic, between 2020 and 2022, significant increases were observed in symptoms, signs, and abnormal clinical and laboratory findings, NEC (R00–R99), and provisional assignment of new diseases of uncertain etiology or emergency use (U00–U18). This means that R00–R99 in the ICD-10 code comprises fever (R50) and headache (R51) associated with COVID-19. Therefore, these increases due to COVID-19 are believed to be disease-induced (26).

Third, the cause of death related to COVID-19 in people with disabilities after COVID-19 was confirmed through ICD-10 code changes. The incidence of intestinal infectious diseases and tuberculosis, and certain infectious and parasitic diseases (A00–B99) decreased after COVID-19. This shows that continuous disease monitoring, prevention strategies, relevant knowledge, and health education following the COVID-19 epidemic can play a role in preventing and controlling intestinal infections associated with COVID-19 (27). In a previous study, the daily notification of tuberculosis outbreaks decreased by 30% during the COVID-19 pandemic (28), which aligns with the findings of this study.

In this study, the incidence of respiratory system diseases (J00–J99), including influenza and pneumonia (J09–J18) and other acute lower respiratory infections (J20–J22), decreased after COVID-19. The symptoms and signs of pulmonary tuberculosis are similar to those of respiratory virus infections, including COVID-19; thus, tuberculosis diagnosis might have been delayed during the COVID-19 pandemic (29). Additional observations are required to confirm whether the decline in tuberculosis diagnoses and intestinal infections continues after 2022, and further research on the causal relationship is required. Overall, the seasonal duration of influenza was shortened with COVID-19 prevention and control, and the positivity rate of influenza decreased significantly within 7–12 weeks in 2020 (28, 30). Generally, it was confirmed that respiratory infectious diseases decreased during the COVID-19 pandemic (28–31). In this study, the incidence of lung diseases due to external agents (J60–J70), other respiratory diseases principally affecting the interstitium (J80–J84), and other diseases of the pleura (J90–J94) increased during COVID-19. The mortality rate of respiratory diseases in Spain did not return to pre-pandemic levels in 2021 and was still high at 30.3% (95% CI 30.2–30.4) compared with the levels in 2019 (29, 31).

The study revealed that COVID-19 deaths in people with disabilities were associated with a higher risk in females, older adults, and people with severe disabilities. Similar to our findings, the mortality rate for COVID-19 among females with disabilities was 2- to 3-fold higher than that of males with disabilities (32–34). However, another study showed a 1.39-fold higher mortality rate in males with disabilities. In this study, the older the disabled person, the higher the COVID-19 mortality rate. This is consistent with previous studies reporting that people with disabilities over 65 years of age are more than twice as likely to die from COVID-19 (33). Additionally, we found that people with severe disabilities are at a significantly greater risk of dying from COVID-19 than people with mild disabilities; this finding is consistent with reported from previous studies (35, 36).

This study had some limitations. First, the observation period of this study after COVID-19 was short (37, 38). Therefore, a more accurate interpretations can only be obtained when longitudinal observational evidence has accumulated in the years to come. Second, this study analyzed data using the middle classification of the ICD-10 codes. Due to the limitations of the information provided by the National Statistical Office in Korea, it was impossible to analyze the ICD-10 codes further divided into sub- and detailed classifications. Consequently, we could only observe the comprehensive status of the investigation rate and cause of death related to COVID-19. Third, we did not conduct a detailed analysis of COVID-19-related diseases. Through a detailed analysis related to COVID-19, strategies to respond to diseases in the event of a new infectious disease, as well as COVID-19 itself, are required. However, through this study, the death patterns of people with disabilities in Korea after COVID-19 were broadly confirmed. However, it is also necessary to reveal the differences in the 15 types of disabilities in Korea, and international comparisons are also required.

This study sought to identify differences in patterns of causes of death for people with disabilities in South Korea before and during COVID-19. Accordingly, we aimed to comprehensively confirm the trend of the cause of death of people with disabilities from 2017 to 2022. Additionally, we confirmed differences in the COVID-19 mortality rate for people with disabilities by sex, age, and degree of disability. However, it is difficult to explain how COVID-19 affected people with disabilities and what factors put them at a higher risk compared with the general population. Therefore, these points will need to be addressed in future research.

## 5 Conclusions

Our findings revealed that the mortality rate among people with disabilities significantly increased during the COVID-19 pandemic compared with that from before the pandemic, and the rate was higher than that in the general population. Considering this high mortality rate among people with disabilities, it is important to address the attendant poor health risk factors to reduce the COVID-19 mortality gap between people with disabilities and the general population. Additionally, short- and long-term public health interventions addressing COVID-19 risks are required.

## Data availability statement

The original contributions presented in the study are included in the article/supplementary material, further inquiries can be directed to the corresponding author.

## Author contributions

Y-SK: Conceptualization, Supervision, Validation, Writing – original draft, Writing – review & editing. Ju-HK: Data curation, Validation, Methodology, Writing – original draft. SK: Formal analysis, Software, Writing – original draft. Jo-HK: Formal analysis, Software, Writing – original draft. H-JK: Validation, Visualization, Writing – original draft. SH: Conceptualization, Funding acquisition, Investigation, Project administration, Writing – original draft.
